# A Rhythm-Based Authentication Scheme for Smart Media Devices

**DOI:** 10.1155/2014/781014

**Published:** 2014-07-07

**Authors:** Jae Dong Lee, Young-Sik Jeong, Jong Hyuk Park

**Affiliations:** ^1^Department of Computer Science and Engineering, Seoul National University of Science and Technology, Seoul 139-743, Republic of Korea; ^2^Department of Multimedia Engineering, Dongguk University, Seoul 100-715, Republic of Korea

## Abstract

In recent years, ubiquitous computing has been rapidly emerged in our lives and extensive studies have been conducted in a variety of areas related to smart devices, such as tablets, smartphones, smart TVs, smart refrigerators, and smart media devices, as a measure for realizing the ubiquitous computing. In particular, smartphones have significantly evolved from the traditional feature phones. Increasingly higher-end smartphone models that can perform a range of functions are now available. Smart devices have become widely popular since they provide high efficiency and great convenience for not only private daily activities but also business endeavors. Rapid advancements have been achieved in smart device technologies to improve the end users' convenience. Consequently, many people increasingly rely on smart devices to store their valuable and important data. With this increasing dependence, an important aspect that must be addressed is security issues. Leaking of private information or sensitive business data due to loss or theft of smart devices could result in exorbitant damage. To mitigate these security threats, basic embedded locking features are provided in smart devices. However, these locking features are vulnerable. In this paper, an original security-locking scheme using a rhythm-based locking system (RLS) is proposed to overcome the existing security problems of smart devices. RLS is a user-authenticated system that addresses vulnerability issues in the existing locking features and provides secure confidentiality in addition to convenience.

## 1. Introduction

Recently, extensive studies have been conducted on smart devices with touch screens in various fields. Some of the examples of smart devices with touch screens include tablets, smartphones, smart TVs, smart refrigerators, and smart media devices. In particular, a smartphone is a representative example of the capability of smart devices to provide a range of functionality despite device miniaturization. This has happened because smartphones continuously evolve as more smartphones with advanced performance capabilities are introduced in the market. Smart devices provide not only several basic functions such as a telephone, alarm clock, notes, schedule, and health management but also additional entertainment features such as books, movies, music, and shopping. They also provide various business functions such as mobile office, real-time SNS, and payment manager to improve business efficiency, and, in particular, big data processing based on smart devices with mobile cloud computing infrastructure. Although miniaturization and the lightweight feature of smart devices can provide users with the convenience of portability, smart device has potential risks of being lost or stolen [1–9]. Accordingly, a countermeasure to mitigate risks on smart devices loss or theft is required now more than ever. Smart devices also have critical data. Hence, they expose users to potential losses due to data leakage and malicious attacks. To protect confidential data, smart devices provide many forms of locking features such as drag, motion, pattern, password, personal identification number (PIN), and face, fingerprint, or a combination of face and voice recognition. However, they are less secure and highly vulnerable to shoulder surfing or smudge attacks [10–16].

In this paper, a novel locking scheme called rhythm locking system (RLS) is proposed to provide a convenient locking activity using rhythm while overcoming the vulnerability of basic locking functions. RLS is a user authentication system that provides secured confidentiality and convenience using unique rhythms set by the user. It also provides a simple interface, thereby enabling easy locking and fast unlocking.

The remainder of this paper is organized as follows. In Section 2, security authentication systems and basic embedded locking features are discussed. In Section 3, the locking scheme of RLS, proposed in this paper, is explained. Next, in Section 4, the design of RLS is detailed and its implementation is described in Section 5, followed by its performance evaluation in Section 6. Finally, the conclusion and future research activities are described in Section 7.

## 2. Related Works

In this section, we discuss the basic embedded locking features used in smart devices and various secure authentication systems. Security authentication systems and their descriptions are summarized in Table 1. Basic locking features embedded in smart devices are summarized in Table 2.

## 3. Locking Scheme of RLS

### 3.1. Key Generation

**Figure 1:**
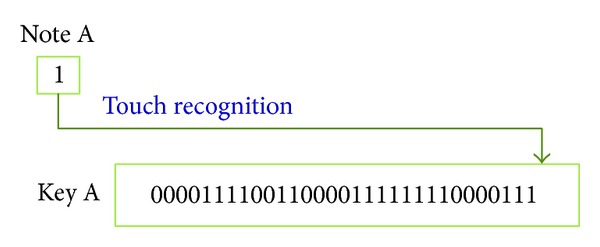
Generation of a single key.

In this paper, we propose a RLS which uses touch rhythm as a secret pattern in a smart media device, which is dependent on auditory and behavior memory. The RLS receives a touch rhythm via a touch screen from a user and defines a track to record this rhythm. At the same time, unit time is measured for tracks. The measurement period ranges from first touching time to the configured time.  Figure 1 shows a single key created for a touch button called A.

**Figure 2:**
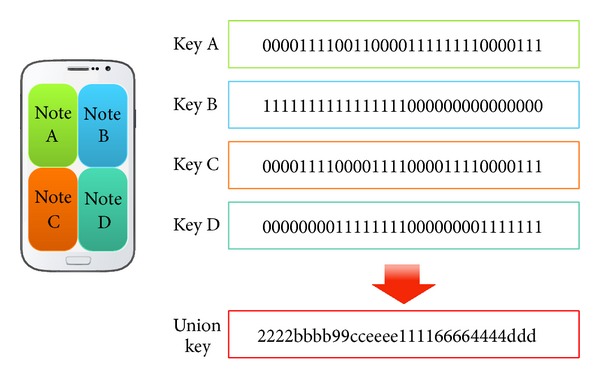
Generation of a union key using a single track for each key.

Figure 2 shows the generation of a union key using multiple tracks to increase the complexity of the secret pattern stored internally. A union key for tracks is generated through the touch recognition of four buttons, A, B, C, and D, using the matching table summarized in Table 3.

This method is distinctively different from the existing button pressing password setup. The available number of rhythm patterns for key generation increases exponentially depending on the time precision setup. Thus, even if malicious users know the positions of the buttons, it is very difficult to infer a user's unique rhythm pattern.

### 3.2. Authentication Process

**Figure 3:**
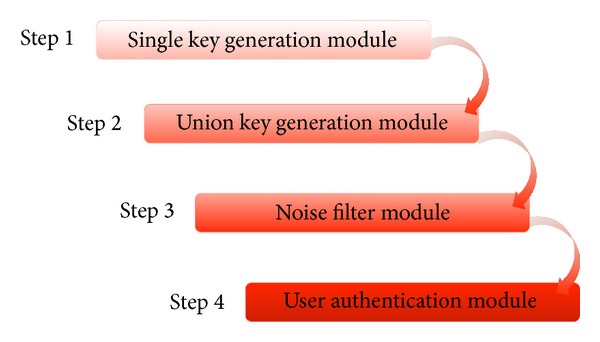
RLS authentication process.

The RLS authentication consists of four steps conducted using four modules.  Figure 3 shows the RLS authentication process. In Step 1, a single key is generated through user input value from the interface. In Step 2, a single union key is generated from the keys generated in Step 1. In Step 3, after the key generation process, improper noise is filtered for authentication. In Step 4, authentication is performed by comparing the stored rhythm pattern with a noise-filtered union key from Step 3.

## 4. Design of RLS

**Figure 4:**
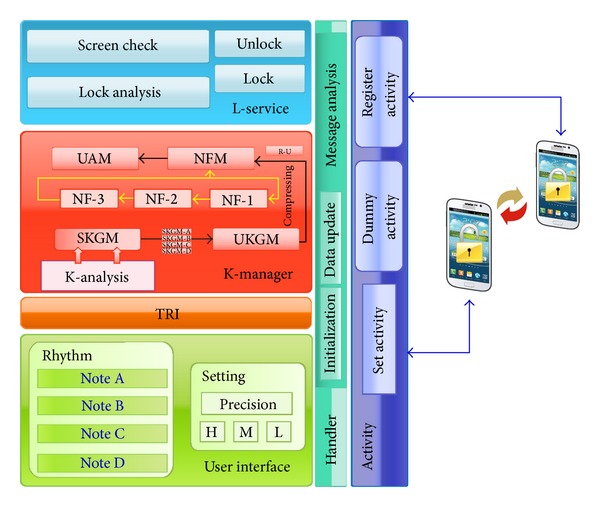
Architecture of RLS.

The RLS consists of six main components. The first component is largely in terms of functionality and the second is the user interface. The third component is the time resolution inspector (TRI) that measures time precision of the RLS. The fourth component is a key manager (K-manager) that manages the entered rhythm patterns. The fifth component is a lock service (L-service) that maintains and manages the locking service of the RLS, and the final component is a handler that delivers information for the visualization of activities.  Figure 4 shows the overall architecture of the RLS.

The* user interface* component is divided into two modules, that is, rhythm and setting. Rhythm consists of four interfaces, Note A, Note B, Note C, and Note D, to set up rhythm patterns from the user. Settings are configured to select one of the three levels, high (H), medium (M), and low (L), depending on the acceptable error range and precision that are set for rhythm pattern input.

*TRI *measures the time interval for which the input is detected, according to the time precision set in the user interface. These measured values consist of a pair of note types and times followed by the inputs of Note A, Note B, Note C, and Note D, which are transferred to a K-manager.

*K-manager *consists of four modules, that are, key analysis (K-analysis), single key generation module (SKGM), union key generation module (UKGM), noise filter module (NFM), and user authentication module (UAM). K-analysis analyzes a pair of data received from the TRI and classifies them according to the note type. SKGM converts a classified note from K-analysis into a single key. UKGM transforms the converted single key from notes to a single complex union key. NFM performs filtering in three steps, NF-1, NF-2, and NF-3, with respect to the raw union key (R-U), converted in the UKGM, according to the acceptable error range set in the user interface. UAM performs either confirmation, when rhythm pattern authentication is set up, or comparison with the existing rhythm patterns to unlock the system when the RLS is executed on a smart device.

*L-service *consists of a screen check that provides a screen according to execution of the RLS operation, a lock analysis that analyzes a locking status, a lock that starts the RLS, and an unlock that stops the RLS.

*Handler* is responsible for delivering data synchronization and control messages between activity and user interface and between activity and L-service. Message analysis, in handler, analyzes received data and delivers visual information about the activity.

*Activity* consists of the following modules: register activity for running the RLS by receiving the rhythm pattern values from a user; dummy activity for visualization while the RLS is running; and set activity for input, confirmation of time precision of the RLS, and other setup activities of a user.

## 5. Implementation of the RLS

**Figure 5:**
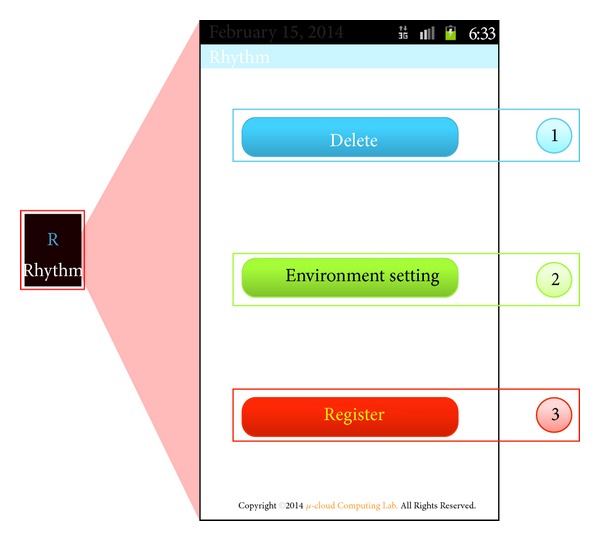
Initial screen of the RLS.

The initial screen of the RLS proposed in this paper is shown in Figure 5. Pressing ①, as shown in Figure 5, deletes a previously set rhythm pattern, while pressing ② moves to activity, by which a user can set the noise and precision of rhythm patterns. Pressing ③ moves to activity, by which the user's unique rhythm pattern can be registered.

**Figure 6:**
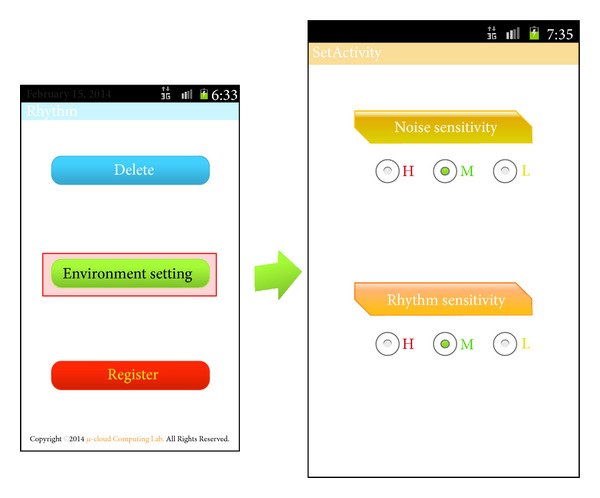
Screen of noise and rhythm setup for the RLS.

Figure 6 shows the setup of activity, which sets an acceptable error of noise and rhythm of the RLS. For noise sensitivity, a level of H, M, and L can be selected according to the filtering level of noise. For rhythm sensitivity, a level of H, M, and L can be selected according to the acceptable error range, which is recognized when a user enters a rhythm. Setup values selected in noise and rhythm sensitivity are applied to the input sensitivity for setting up rhythm patterns and unlocking the screen when the RLS is running. The default setup value is M for both noise and rhythm sensitivity.

**Figure 7:**
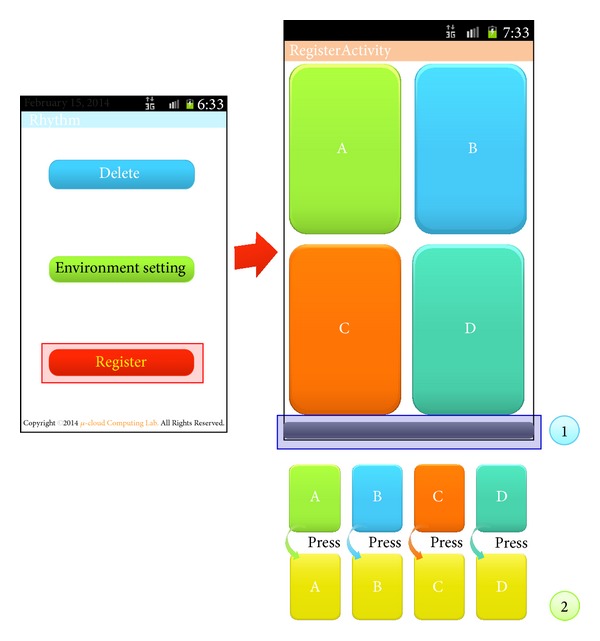
Register activity of the RLS.

Figure 7 shows a screen for input of rhythm patterns to execute the RLS. In Figure 7, ① is the register activity, which consists of Note A, Note B, Note C, and Note D. ② displays the success or failure of recognition in the system when a user enters a rhythm on Note A, Note B, Note C, or Note D. Here, a blue-colored timer progress bar is shown.

**Figure 8:**
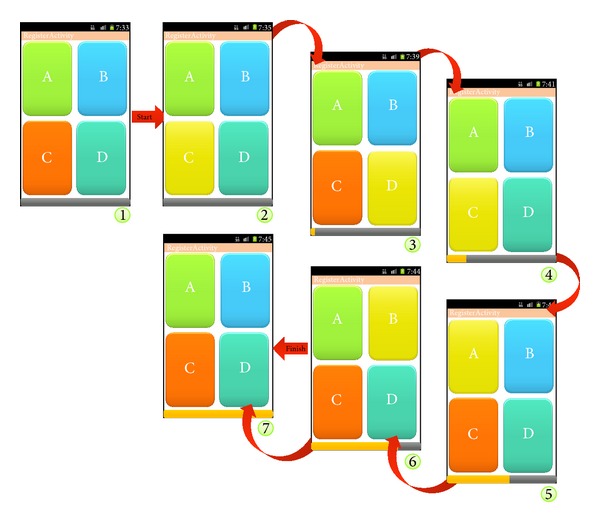
Rhythm pattern setup process in the RLS.

Figure 8 shows rhythm inputs of C, D, C, A, and B in order as entered by a user. In Figure 8, ① shows the status that initial input has not been detected, while ② shows that a timer progress bar is displayed as input C is recognized, and ③ shows the status that input D is recognized while a timer progress bar is running. As such, the timer progress bar runs independently while the initial input is recognized. While the timer progress bar is running, each single key for A, B, C, and D is internally generated. Once the timer progress bar terminates, single keys entered up to now are composed into a union key. Since the RLS adds not only the physical Interfaces A, B, C, and D entered by a user but also the logical time, it provides an enhanced security functionality.

## 6. Performance Evaluation

### 6.1. Evaluation of Security Strength

We conducted an experiment using a prototype of our proposed scheme. The prototype was developed on Android 4.3 Jelly Bean. In the experiment, we used a smart media device with a Qualcomm Snapdragon 800 2.3 GHz CPU and DDR3 3 GB RAM.

We performed experiments to determine the false acceptance rate (FAR) and false rejection rate (FRR) of the RLS to evaluate its security strength. The standard keys for FAR and FRR are shown in Table 4. For FAR, an arbitrary control key with the same length as the original key was created to perform the comparison. For FRR, an arbitrary control key was created by considering falsely rejected circumstances to perform the comparison. In Key 1, 85/1:10/0:5/2:10/0:5/3:10/0:5/2:10/0:5/3:10/0:5/4:10, in Table 4, “85” refers to a key length. Next, 1:10 indicates that Interface A, in Figure 7, was entered 10 s after input began, while 1 refers to the converted value obtained from the matching table. That is, the first number, 1, indicates the entered interface, while the following number, 10, after colon, refers to the time which elapsed while the input is received.

**Figure 9:**
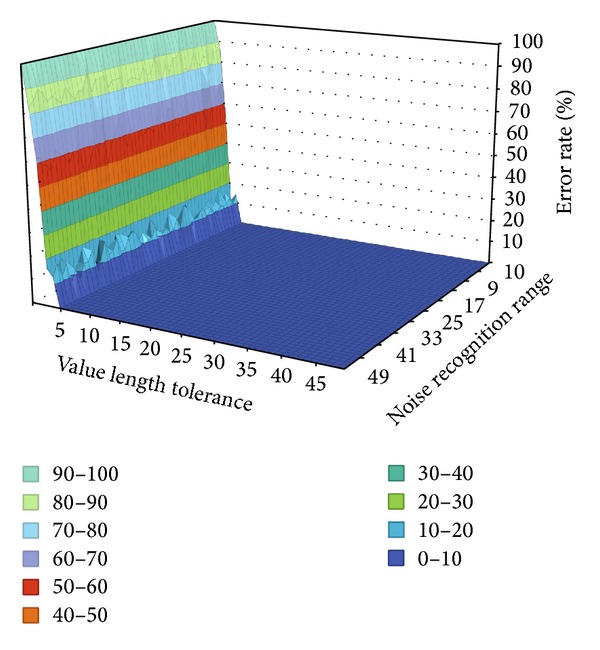
FAR and FRR performance of Key 1.

Figure 9 shows a graph of FAR and FRR with Key 1 in Table 4. As the value length tolerance in the lower left area becomes larger, the allowable error range becomes less when the length of each interface is examined. As the noise recognition range in the lower right area becomes larger, false recognition due to noise becomes more frequent. As numbers with respect to these two increase sequentially, an error rate is calculated by comparing the control key, which is created dynamically, and the standard key. As shown in Figure 9, a 0% error rate was obtained irrespective of the effect of the value length tolerance and the noise recognition range.

**Figure 10:**
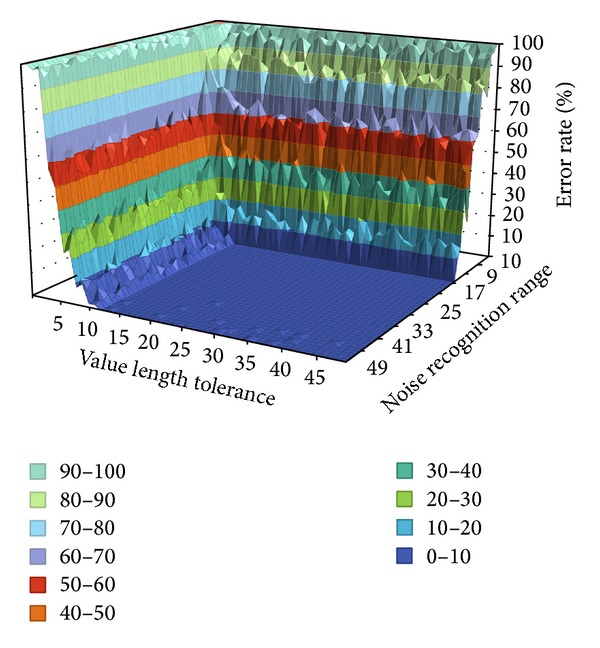
FAR and FRR performance of Key 2.

Figure 10 shows a graph of FAR and FRR with Key 2 in Table 4. It is configured in the same manner as in Figure 9, reaffirming that error rate decreases as the allowable range of the value length tolerance becomes smaller with respect to Key 2. It also shows that, as the noise recognition range becomes larger, error rate becomes smaller.

**Figure 11:**
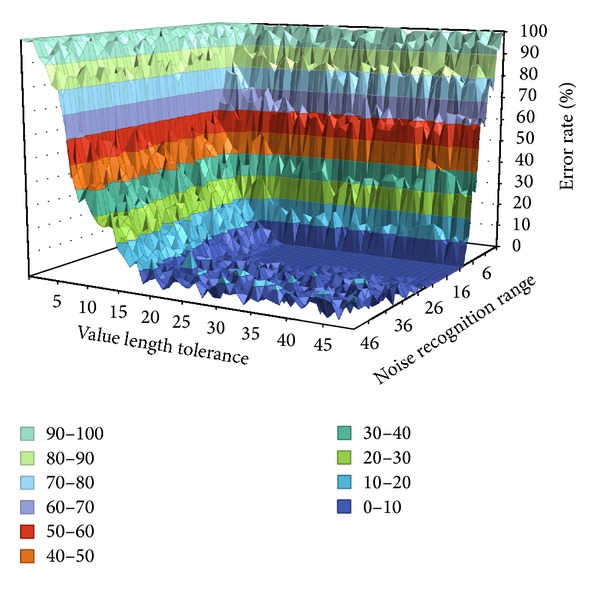
FAR and FRR performance of Key 3.

Figure 11 shows a graph of FAR and FRR with Key 3 in Table 4. As with Figure 10, as the value length tolerance and noise recognition range become larger, the error rate becomes smaller. Thus, if a user sets a rhythm pattern of the RLS to one more than a specific threshold value, strong security can be ensured.

### 6.2. Comparison with Existing Locking Schemes

In this section, existing locking schemes such as pattern lock, PIN, and password are compared with the RLS proposed in this paper, with respect to various attacking techniques.

Against a brute force attack, the number of patterns that can be set for locking determines security strength. PIN provides relatively weak security compared to other locking schemes, because it has a limited input length as well as the restriction that only a number can be used. Pattern lock has an advantage in terms of input of various patterns; it provides security stronger than PIN but weaker than password and the proposed RLS. Password and the RLS have similar security strength against brute force attacks.

The shoulder surfing attack begins when a user enters a pattern to unlock the screen. Pattern lock, which uses various patterns but is vulnerable to visual memory, and PIN, which uses fixed arrangement of numbers, both, therefore, provide weak security. Password is robust against the shoulder surfing attack owing to the large number of possible patterns. The RLS also has robust security, with a rhythm-based locking scheme using a logical time.

The dictionary attack is a method that employs all meaningful words or sentences in a dictionary. The pattern lock and the RLS provide robust security against dictionary attack because they use an entirely different method to set the locking pattern. However, PIN and password are moderately vulnerable because a user may employ meaningful numbers, symbols, or words.

The smudge attack uses a simple trace to discern a locking pattern. A trace is deployed to infer a locking pattern while the user's input is entered to unlock the screen. Pattern lock and PIN show weak security because of easy collection of trace owing to the fixed arrangement on the screen. If a password is set with a long and complicated pattern, it can provide robust security; however, if it is set with a short and simple pattern, it provides weak security. The RLS provides robust security against smudge attacks because it combines physical and logical schemes.

Table 5 shows relative security of the existing locking systems and the RLS against various attack methods. The proposed RLS displays the strongest security against the brute force attack, shoulder surfing attack, dictionary attack, and smudge attack, which are some of the widely used attacks for touch-screen-based smart devices.

## 7. Conclusion

Smart media devices provide users with a variety of functions leading to their wide use. Most vendors have developed a variety of functions to provide better services to the end users. In particular, smart media devices have made significant advancement in terms of weight reduction, miniaturization, and various functions offered, but the most basic security issues have been ignored. As a result, although many basic locking features are embedded, smart media devices are vulnerable to a number of attacks.

In this paper, we proposed a rhythm-based locking system which considers rhythm as logical behavior. The proposed system provides not only strong security against malicious attackers but also convenience of memory to users. In addition, it is composed of a simple interface structure so that all ages can use it conveniently. Even if pressing positions are exposed, it is extremely difficult to predict precise timings, thereby ensuring high security strength.

In the future, a user authentication structure utilizing various sensors embedded in smart media devices will be studied. Stronger locking functions will be provided by considering the angle or the number of slopes. A unique type of user authentication system structured through unique recognition media will also be researched.

## Figures and Tables

**Table 1:** Security authentication systems and their descriptions.

Security authentication system	Description
Something you know	This authentication mode relies on an end user's memory. That is, this mode depends on an individual's memory. In general, users refer to personal information when setting a secret key. Although it has the easy-to-remember advantage, it is vulnerable because malicious attackers can easily take advantage. It can incur additional damage due to leakage of the key as the authentication process can be exposed because of user carelessness. Using this method, a user must memorize the key. If a user forgets the secret key, even a rightful user cannot access the system or services.

Something you have	This authentication mode uses object(s) that a user owns. For example, objects such as barcodes, QR codes, magnetics, and RFIDs are used. That is, this mode depends on the object(s) that a user possesses. If the object is possessed always, this mode provides convenience of authentication and relatively less leakage risk than something you know. However, this method is somewhat inconvenient as the user must always possess the object. If the object is lost or stolen, additional damage can be incurred from malicious attackers. Further, if the object is damaged, a rightful user cannot access the system or services.

Something you are	This authentication mode uses biometric information. This mode uses two different types of techniques: (a) recognition of physiological information and (b) recognition of behavior patterns. A scheme of recognizing physiological information uses individual characteristics of the user. For example, fingerprint recognition, iris recognition, vein recognition, face recognition, voice recognition, and palm print recognition are used. That is, this mode depends on a user's unique biological characteristics. Identity theft by malicious attackers is nearly impossible, and a risk of loss or change is extremely low, which is important for security. A prerequisite for such a method is to have very high recognition precision. If recognition rate is low, authentication of malicious attackers, who have similar or mimicked personal characteristics, can successfully gain access to the system.

**Table 2:** Basic locking features embedded in smart devices.

Locking system	Description
Pattern lock	This authentication system uses end user's visual memory. Using nine points in a three-by-three grid, a user creates a drag pattern. This method belongs not only to the something you know category, which is based on memory, but also to the behavior pattern recognition category, since it utilizes finger motion memory. The number of available secret patterns in this system is 388,912, which is relatively small due to the limited and fixed arrangement of the nine possible points. This method can be vulnerable to a brute force attack if a user creates a drag pattern using a fewer than suggested number of points to unlock the screen faster. It is also vulnerable to a shoulder surfing attack by malicious attackers due to the visual aspects of a drag pattern. Finally, it is vulnerable to the smudge attack as well, which uses the characteristics of touch screen, in case of theft.

Face recognition	This authentication mode uses biometric information. This method depends on the camera in smart devices and has the advantage of requiring additional memory or management of the locking key due to unique characteristics. However, unlocking the locked screen could be difficult not only due to low performance of the embedded camera but also due to environmental factors (e.g., face recognition range can be limited because of the amount of ambient light). Further, it is vulnerable to the application of similar faces or recognition using photos and videos, which is why this method, in general, is rarely used.

Password	This authentication system uses visual memory and is familiar to most users. Passwords are used by offering a virtual keypad where a combination of alphabetic, numeric, and special characters can be used. Security is dependent on the strength of the password, which depends on the combination of chosen characters. If a password is considerably short in length, for quicker screen unlocking, then it could be vulnerable to a smudge or shoulder surfing attack. If the length of password is considerably long, the user may experience password memory loss possibly due to confusion. Password is also vulnerable to the dictionary attack if the attacker has access to the user's personal information.

PIN	This authentication system has yet to overcome confusion due to the complicated combination of characters used for a password. PIN uses only a numerical value from 0 to 9. Further, it uses combinations of only four numbers so that the available PIN count is less than 10,000, which is considerably small. Thus, it is vulnerable to a brute force attack. It is also vulnerable to shoulder surfing and smudge attacks due to generated traces and the visual nature of pressing four numbers.

**Table 3:** Matching table for generation of union key.

Key combination	Union note
Non	0
A	1
B	2
C	3
D	4
A + B	5
A + C	6
A + D	7
B + C	8
B + D	9
C + D	a
A + B + C	b
A + B + D	c
A + C + D	d
B + C + D	e
A + B + C + D	f

**Table 4:** Three types of the standard key based on key length.

Type	Rhythm locking pattern	Locking pattern characteristics
Key 1	85/1:10/0:5/2:10/0:5/3:10/0:5/2:10/0:5/3:10/0:5/4:10	(i) Short length (ii) Six single notes (iii) Simple rhythm

Key 2	158/3:20/0:3/2:20/0:3/5:20/0:3/2:20/0:3/3:20/0:3/5:20/0:3/2:20	(i) Medium length (ii) Two composite notes (iii) Six single notes (iv) Simple rhythm

Key 3	220/1:50/0:5/5:10/0:5/2:40/0:15/3:30/0:5/a:60	(i) Long length (ii) Two composite notes (iii) Three single notes (iv) Irregular rhythm

**Table 5:** Comparison of the existing locking systems and the RLS against various attacks.

	Pattern lock	PIN	Password	RLS
Brute force attack	△	X	O	O
Shoulder surfing attack	X	X	O	O
Dictionary attack	△	X	△	O
Smudge attack	X	X	△	O

O: strong, △: medium, and X: weak.
